# Methylpiperidinium Iodides as Novel Antagonists for α7 Nicotinic Acetylcholine Receptors

**DOI:** 10.3389/fphar.2018.00744

**Published:** 2018-07-10

**Authors:** Jhon J. López, Jesús García-Colunga, Edwin G. Pérez, Angélica Fierro

**Affiliations:** ^1^Department of Chemistry, Faculty of Sciences, University of Chile, Santiago, Chile; ^2^Departamento de Neurobiología Celular y Molecular, Instituto de Neurobiología, Universidad Nacional Autónoma de México, Mexico City, Mexico; ^3^Department of Organic Chemistry, Faculty of Chemistry, Pontificia Universidad Católica de Chile, Santiago, Chile

**Keywords:** nicotinic acetylcholine receptors, nicotinic antagonists, methylpiperidinium iodides, molecular ligand–receptor interactions, the whole-cell voltage-clamp technique, molecular docking, molecular dynamics

## Abstract

The α7 nicotinic acetylcholine receptor (nAChR) is expressed in neuronal and non-neuronal cells and is involved in several physiopathological processes, and is thus an important drug target. We have designed and synthesized novel piperidine derivatives as α7 nAChR antagonists. Thus, we describe here a new series of 1-[2-(4-alkoxy-phenoxy-ethyl)]piperidines and 1-[2-(4-alkyloxy-phenoxy-ethyl)]-1-methylpiperidinium iodides (compounds **11a-11c** and **12a-12c**), and their actions on α7 nAChRs. The pharmacological activity of these compounds was studied in rat CA1 hippocampal interneurons by using the whole-cell voltage-clamp technique. Inhibition of the choline-induced current was less for **11a-11c** than for the methylpiperidinium iodides **12a-12c** and depended on the length of the aliphatic chain. Those compounds showing strong effects were studied further using molecular docking and molecular dynamics simulations. The strongest and non-voltage dependent antagonism was shown by **12a**, which could establish cation–π interactions with the principal (+)-side and van der Waals interactions with the complementary (-)-side in the α7 nAChRs. Furthermore, compound **11a** forms hydrogen bonds with residue Q115 of the complementary (-)-side through water molecules without forming cation–π interactions. Our findings have led to the establishment of a new family of antagonists that interact with the agonist binding cavity of the α7 nAChR, which represent a promising new class of compounds for the treatment of pathologies where these receptors need to be negatively modulated, including neuropsychiatric disorders as well as different types of cancer.

## Introduction

Nicotinic acetylcholine receptors (nAChRs) are membrane proteins belonging to the superfamily of Cys-loop ligand-gated ion channels that include serotonin (5-HT_3_), GABA_A_, GABA_C_, and glycine receptors (Lester et al., 2004; Dineley et al., 2015). They are pentameric structures composed of four different subunits in muscle receptors (α1, β1, γ or ε, and δ), whereas the neuronal subtypes are assembled as homomeric or heteromeric receptors from diverse combinations of subunits (α2-α10 and β2-β4) conferring different physiological, pharmacological and biophysical properties (Gotti et al., 2009; Blum et al., 2010; Zoli et al., 2017).

The agonist binding site, known as the “aromatic box,” is located at the interface between two α subunits [residues Y89, W143, Y185, Y192 of the principal (+)-side and W53 of the complementary (-)-side]; or between one α subunit [principal (+)-side] and one β subunit [complementary (-)-side, W53] (Olsen et al., 2014). Heteromeric nAChRs with two α subunits exhibit two binding sites for the agonist, whereas homomeric receptors have five identical binding sites for the agonist (Albuquerque et al., 2009; Gotti et al., 2009).

Of all nAChRs, the α7-subtype is one of the most abundant in the nervous system and is involved in several physiological roles, neuropsychiatry and neurodegenerative diseases (Romanelli et al., 2007; Gotti et al., 2009; Dineley et al., 2015). For instance, increased ACh signaling in hippocampus increases depression-like behavior. In addition, the α7 subunit of nAChR is highly expressed in this region. Thus, under increased ACh conditions, hippocampal α7 nAChRs may contribute to modulation of depression which can be reversed by the α7 nAChR antagonists (Mineur et al., 2017). α7 nAChRs are also present on non-neuronal cells such as bronchial epithelium and keratinocytes (Egleton et al., 2008; Albuquerque et al., 2009; Gahring et al., 2017). On the other hand, tobacco smoking seems to be the chief risk factor for lung, pancreatic, colon, gastric, and bladder cancers (Cooke and Bitterman, 2004; Wong et al., 2007; Egleton et al., 2008), suggesting that nicotine contribute to their pathophysiology. In this regard, several studies have shown that α7 nAChRs are involved in nicotine-induced proliferation of normal human bronchial epithelial cells, as well as cell proliferation in small cell lung cancer and non-small cell lung cancer, in which α7 nAChR antagonists decreased these nicotine effects (Heeschen et al., 2002; Trombino et al., 2004; Dasgupta et al., 2006). For these reasons, the α7 nAChR has been considered as potential pharmacological target, and in the last decades a variety of selective ligands have been synthesized; however, only few compounds are clinically used (Dunbar et al., 2011; Mazurov et al., 2011; Terry et al., 2015). In this sense, the development of selective antagonists for α7 might be alternative for the treatment of several cancers (Dwoskin and Crooks, 2001; Egleton et al., 2008; Innocent et al., 2008; Peng et al., 2010).

**FIGURE 1:**
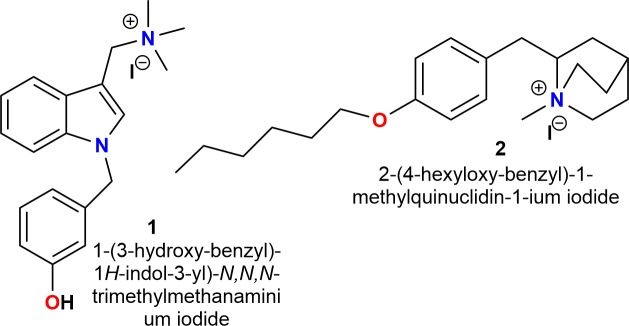
Chemical structures of selective antagonists for α7 nAChRs (Arias et al., 2013; Pérez et al., 2013; López et al., 2015).

Recently, our group developed the selective antagonists for α7 nAChRs compounds **1** and **2** (Arias et al., 2013; Pérez et al., 2013; López et al., 2015; **Figure 1**).

**FIGURE 2:**
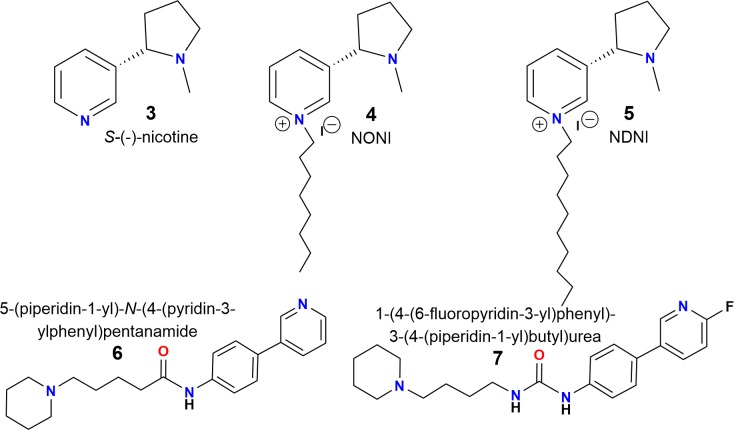
Chemical structures of *S*-(-)-nicotine (**3**), *N-n*-alkylnicotinium analogs NONI (**4**) and NDNI (**5**) and piperidine derivatives (**6**) and (**7**) (Crooks et al., 1995; Roy et al., 1999; Wilkins et al., 2002).

There are two important criteria for generating α7 nAChR antagonists: (a) a center containing a nitrogen atom with a positive charge (cationic center), which can interact with the “aromatic box” of the nAChR. In this regard, the pyridinic nitrogen atom of nicotine (**Figure 2**, compound **3**), alkylated with long aliphatic chains (3–9 atoms of carbon), results in a potent and selective antagonist for nAChRs (**Figure 2**, compounds **4** and **5**) (Crooks et al., 1995; Wilkins et al., 2002); (b) and alkyl groups located at the “*para*” position of an aromatic ring, which are important for van der Waals interactions with the amino acid residues of the complementary (-)-side, relevant for the selectivity toward the α7 subtype (Crooks et al., 1995; Arias et al., 2013; Pérez et al., 2013).

**FIGURE 3:**
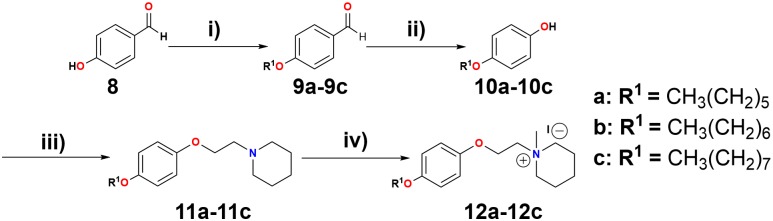
Reagents and conditions: (i) K_2_CO_3_, R^1^-Br, EtOH, reflux, 18 h. (ii) H_2_O_2_ (30%), H_3_BO_3_, THF, H_2_SO_4_. (iii) DIAD, 1,4-dioxane, PPh_3_, 2-piperidinoethanol, reflux in N_2_ atmosphere, 48 h. (iv) CH_3_I, acetone, 16 h.

Additionally, a simple piperidine ring has been used for generating selective agonists for α7 nAChRs (**Figure 2**, compounds **6** and **7**) (Ghiron et al., 2010). It is important to mention that the design, synthesis and chemically characterized of new piperidine derivatives (**Figure 3**, **11a**-**11c** and **12a**-**12c**) was based on the binding cavity and our main goal was to evaluate a new chemical architecture as potential ligands for the α7 nAChR. Additionally, their pharmacological activity was tested in native hippocampal α7 nAChRs, and their interaction with the receptor was studied using molecular docking and molecular dynamics simulations. In summary, these compounds have a piperidine ring (*N*-methylated or not) and include an aromatic ring substituted at the “*para*” position with alkyl groups containing 6–8 carbon atoms, expected to enhance the selectivity for α7 nAChRs (Arias et al., 2013; López et al., 2015).

## Materials and Methods

### Chemical Synthesis

For the chemical synthesis of new piperidine derivatives, commercial reagents, and solvents were used (Sigma-Aldrich and MERCK), employing previously described synthetic methodologies (Roy et al., 1999; Peters et al., 2006; Pérez et al., 2012, 2013; Arias et al., 2013; Dang et al., 2016).

### Chemical Characterization

^1^H and ^13^C nuclear magnetic resonance (NMR) spectra were recorded at 200 or 400 MHz and 50 or 100 MHz for ^1^H and ^13^C respectively, on Bruker ACP-200 or Bruker Avance 400 spectrometers. The results are detailed in the Supplemental Information. Chemical shifts are reported in δ values [parts per million (ppm)] relative to an internal standard of tetramethylsilane in deuterated chloroform (CDCl_3_) or deuterated methanol (CD_3_OD), and coupling constants (*J*) are given in Hertz. Precoated silica gel 60 plates (Merck 60 F_254_ 0.2 mm) were used for thin-layer chromatography and silica gel 60 (0.015–0.040 mm) for column chromatography. Spots on thin-layer chromatograms were visualized by spraying with Dragendorff’s reagent or under ultraviolet light at 254 nm.

### Electrophysiological Recordings

All experimental procedures were carried out in accordance with the National Institute of Health Guide for Care and Use of Laboratory Animals and were approved by the Institutional Animal Care Committee of the Universidad Nacional Autónoma de México, with an effort to minimize the number of animals used and their suffering. The experiments were performed as previously described (Vázquez-Gómez et al., 2014; López et al., 2015). Briefly, brain slices were obtained from Sprague Dawley rats, 13–16 days after birth. Rats were deeply anesthetized with isoflurane and decapitated. Their brains were removed and placed into an ice cold (4°C) solution containing (in mM): 250 sucrose, 2.5 KCl, 1.2 NaH_2_PO_4_, 5 MgCl_2_, 0.5 CaCl_2_, 26 NaHCO_3_, 10 glucose, pH 7.4. Coronal slices of 350 μm thickness containing the hippocampal CA1 area were cut with a Vibratome Leica VT 1000 and submerged in artificial cerebrospinal fluid containing (in mM): 125 NaCl, 2.5 KCl, 1.23 NaH_2_PO_4_, 1 MgCl_2_, 2 CaCl_2_, 26 NaHCO_3_, 10 glucose, pH 7.4. The slices were stabilized in this solution for 1 h before electrical recording. All solutions were continuously bubbled with 95% O_2_ and 5% CO_2_ at room temperature.

One slice was transferred into a chamber and superfused during the experiment with artificial cerebrospinal fluid at a rate of ∼2 mL/min. Whole-cell voltage-clamp recordings were performed with a PC-ONE Patch/Whole Cell Clamp (Dagan Corporation, Minneapolis, MN, United States), using a Digidata 1440A acquisition system driven with pClamp 10 (Molecular Devices, Silicon Valley, CA, United States). Patch-clamp electrodes had a resistance of 3–7 MΩ when filled with the internal solution (in mM): 140 K-gluconate, 10 HEPES, 2 MgCl_2_, 0.5 CaCl_2_, 10 EGTA and 2 MgATP (pH 7.4). Data were stored in a PC using a Digidata 1440A AD converter, at a sampling rate of 10 kHz. Interneurons were visualized using an infrared video-microscopy system (BX51WI, Olympus Instruments, Japan) endowed with an 80x water immersion objective. Recorded interneurons were located in the *stratum radiatum* hippocampal CA1 area, and were maintained at a potential of -70 or -20 mV.

The way to explore the effects of piperidine derivatives was previously described for other substances (Vázquez-Gómez et al., 2014; López et al., 2015). Choline (Ch, 10 mM) puffs (2–5 psi, 500 ms) were applied on interneurons through a fine tip glass micropipette placed ∼10 μm from the recorded cell by using a pneumatic picopump (PV830, WPI, Sarasota, FL, United States). Thus, repeated Ch-puffs were applied at 5 min intervals before, during and after the piperidine derivative was added to the bath solution for ∼10 min. In all the experiments, only one concentration of each compound was tested by cell, due to the long-lasting experiment (more than 1 h), and also just to avoid a carry-over effect. The Ch-induced current (*I*_Ch_) amplitude was measured as a function of recording time. pClamp 10 software was used to measure *I*_Ch_ in the absence or presence of the piperidine derivative. Origin 7 software (Microcal Software, Northhampton, MA, United States) was used to analyze and graph the results. Data are presented as mean ± standard error. Comparison of the two population means was performed by paired Student’s *t*-test; *p* < 0.05 was considered statistically significant.

### Computational Methods

Models of compounds **11a** and **12a** were built using Gaussian03 and partial charges were corrected with Electrostatic Potential methodology. Topology and parameters for the ligands was obtained with the ParamChem server, which used the CHARMM27 force field and database for organic compounds (Frisch et al., 2004; Vanommeslaeghe et al., 2010).

#### Homology Modeling

To construct the extracellular domain of the rat α7 nAChR, the structure of the ACh binding protein (AChBP) from *Aplysia californica* at 2.3 Å resolution was used as a template for homology modeling (code 4DBM in the Protein Data Bank) (Grimster et al., 2012).

The target protein and template were aligned with the Multalin server (Corpet, 1988). Using the software MODELLER 9v12 (Šali and Blundell, 1993), 100 runs were carried out with standard parameters and the outcomes were ranked on the basis of the internal scoring function of the software. The best model was chosen as the target model.

#### Molecular Docking

To study characteristics of the principal protein–ligand interactions, molecular docking of the α7 nAChRs was done using the AutoDock 4.0 software suite. In general terms, grid maps were calculated using the autogrid option and centered on the binding sites. The volumes chosen for the grid maps were made up of 60 × 60 × 60 points, with a grid-point spacing of 0.375 Å. For transmembrane domains, a grid covering the whole region was used instead. The autotors option of the software was used to define the rotating bonds in the ligand. In the Lamarckian genetic algorithm dockings, a number of individuals in a population of 1500, a maximum number of 2.5 × 10^6^ energy evaluations, a maximum number of 27.000 generations, a mutation rate of 0.02, and a cross-over rate of 0.80 were employed. The docked compound complexes were built using the lowest docked-energy binding positions (Morris et al., 1998).

#### Molecular Dynamics Simulations

Each protein–ligand complex was solvated with water model TIP3 and submitted to molecular dynamics simulations for 20 ns using an NPT ensemble. NAMD 2.6 software was used to perform dynamics simulations calculations. Periodic boundary conditions were applied to the system in the three coordinate directions. Pressure of one atmosphere and temperature of 310 K were maintained (Phillips et al., 2005).

## Results

### Chemical Synthesis

The piperidine derivatives, 1-[2-(4-alkyloxy-phenoxy-ethyl)]-1-methylpiperidinium iodides, were synthesized (see **Figure 3**). A Williamson synthesis was initially used to react 4-hydroxybenzaldehyde (**Figure 3**, compound **8**) with different alkyl bromides (1-bromohexane, 1-bromoheptane, and 1-bromooctane), in a basic medium (K_2_CO_3_) under reflux (Arias et al., 2013), to produce the corresponding alkyloxy benzaldehydes (**9a**-**9c**). **9a**-**9c** were subsequently oxidized to their respective alkoxy phenols (**10a**-**10c**) by a Baeyer–Villiger reaction (Roy et al., 1999), using H_2_O_2_ (30%), H_3_BO_3_, THF, and H_2_SO_4_, stirring for 24 h at room temperature. Afterward, the alkoxy phenols were combined with 2-piperidineethanol, using a Mitsunobu reaction (Dang et al., 2016), producing 1-[2-(4-alkyloxy-phenoxy-ethyl)]piperidine derivatives **11a**-**11c**. These were dissolved in acetone and reacted with CH_3_I for 16 h (Pérez et al., 2012), producing 1-[2-(4-alkyloxy-phenoxy-ethyl)]-1-methylpiperidinium iodides **12a**-**12c** (quaternary ammonium salts) (Arias et al., 2013). For more details see General procedures in the Supplemental Information.

All the synthesized compounds (**Figure 3**, intermediate and final) were purified by column chromatography and their structures were confirmed by ^1^H-NMR and ^13^C-NMR analysis.

### Electrophysiological Recordings

Several piperidine derivatives (**Figure 3**, **11a**-**11c**, **12a**-**12c**) were tested for their effects on ion currents elicited by choline (*I*_Ch_; Alkondon et al., 1997) in interneurons from the *stratum radiatum* hippocampal CA1 area, that is, on endogenous rat α7 nAChRs (Liu et al., 2012). As in previous reports, the electrical activity of native hippocampal α7 nAChRs was elicited by applying local puffs of 10 mM Ch, resulting in inward ion currents decaying even in the presence of the agonist, due to receptor desensitization; and these currents were inhibited with methyllycaconitine or α-bungarotoxin (Vázquez-Gómez et al., 2014; López et al., 2015).

**FIGURE 4:**
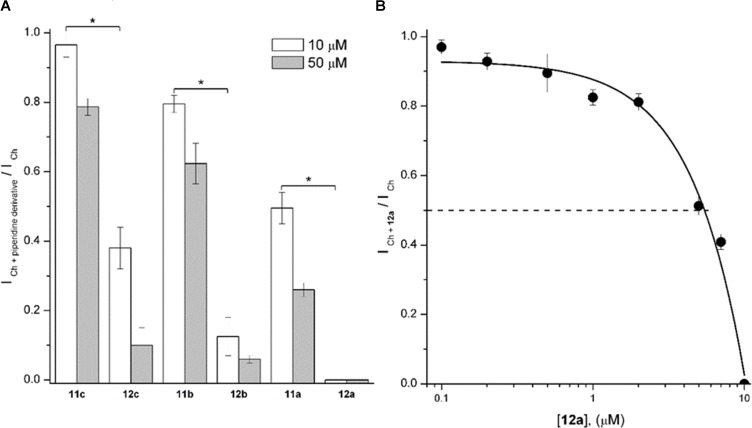
1-[2-(4-Alkyloxy-phenoxy-ethyl)]piperidine (**11a**-**11c**) and 1-[2-(4-alkyloxy-phenoxy-ethyl)]-1-methylpiperidinium iodides (**12a**-**12c**) inhibited rat hippocampal α7 nAChRs. **(A)** The height of the columns represents the ratio between the *I*_Ch_ in the presence of the piperidine derivative (*I*_Ch__+__piperidinederivative_) and the control *I*_Ch_ at 10 (white) and 50 μM (gray columns) of each compound. Experiments were performed as described in Section “Materials and Methods.” Each column represents the mean ± standard error of at least three recorded interneurons. Unless otherwise stated, holding potential was –70 mV. Asterisks indicate significant statistical difference (*p* < 0.05) when compared methylated with non-methylated piperidine derivatives at 10 μM, performed by the Student’s *t*-test. **(B)** The **12a** concentration/Ch-response relation in hippocampal CA1 interneurons. The ratio between the *I*_Ch_ in the presence of **12a** (*I*_Ch__+_ **_12a_**) and the control *I*_Ch_ is plotted as a function of **12a** concentration. Each point is the mean ± standard error from *n* = 3–7 interneurons. Dashed line corresponds to 50% of the maximal control *I*_Ch_.

As an initial screen, 1-[2-(4-alkyloxy-phenoxy-ethyl)] piperidine (**Figure 3**, **11a**-**11c**) and 1-[2-(4-alkyloxy-phenoxy-ethyl)]-1-methylpiperidinium iodides (**Figure 3**, **12a**-**12c**) were tested at 10 and 50 μM. For each concentration, the extent of *I*_Ch_ inhibition depended inversely on the length of the chain, with longer chains producing less *I*_Ch_ inhibition. Furthermore, compounds **12a**-**12c** inhibited the *I*_Ch_ more strongly than the corresponding **11a**-**11c** (**Figure 4A**). Moreover, **11a** was the non-methylated compound exhibiting the highest inhibition; however, the inhibitory potency was minor than the methylated compounds. Thus, the sequence of inhibitory potency was: **12a** > **12b** > **12c** > **11a**. Compound **12a** showed the most potent antagonistic effect on α7 nAChRs: at 10 μM this compound completely inhibited the *I*_Ch_ (**Figure 4A**). For this reason, concentrations of **12a** ranging from 0.2 to 10 μM were tested on *I*_Ch_. The inhibitory effect increased with increasing **12a** concentration (**Figure 4B**), attaining 50% near 5 μM (**Figure 4B**).

**FIGURE 5:**
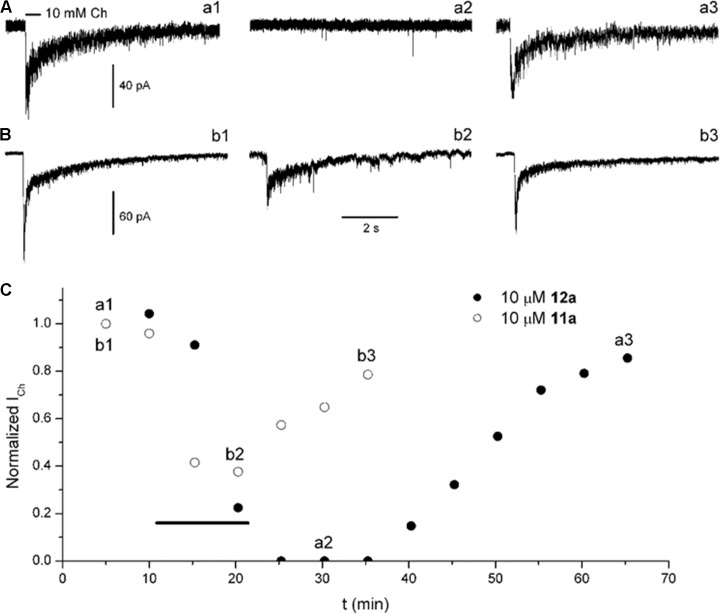
Compounds **12a** and **11a** inhibited rat hippocampal α7 nAChRs. Ch-puffs were applied at 5-min intervals before, during, and after bath application of the corresponding compound to obtain the *I*_Ch_ amplitude as a function of recording time. **(A,B)** Representative *I*_Ch_ determinations from seven (**12a**) and three (**11a**) separate experiments. Ion currents were elicited by a 5-psi, 500-ms puff of 10 mM Ch (line above the record **a1**). The horizontal bar calibration applies for all records. **(C)** The *I*_Ch_ amplitude as a function of time, before, during, and after the application of **12a** (filled circles) and **11a** (open circles). The timing of the corresponding compound application is indicated by the thick black line.

To explore and compare the effects of **11a** and **12a**, Ch-puffs were initially applied at 5-min intervals to obtain the control *I*_Ch_. In the case of **12a**, the *I*_Ch_ was inhibited immediately after application of 10 μM, and the Ch response continued to be completely inhibited after washing out the **12a** (**Figures 5A,C**, record **a2**). The recovery was slow and still incomplete at ∼50 min (**Figures 5A,C**, record **a3**). In the case of **11a**, in contrast, 10 μM moderately inhibited *I*_Ch_ (**Figures 5B,C**, record **b2**) and then the recovery, which was also slow and incomplete, occurred sooner (**Figures 5B,C**, record **b3**).

**FIGURE 6:**
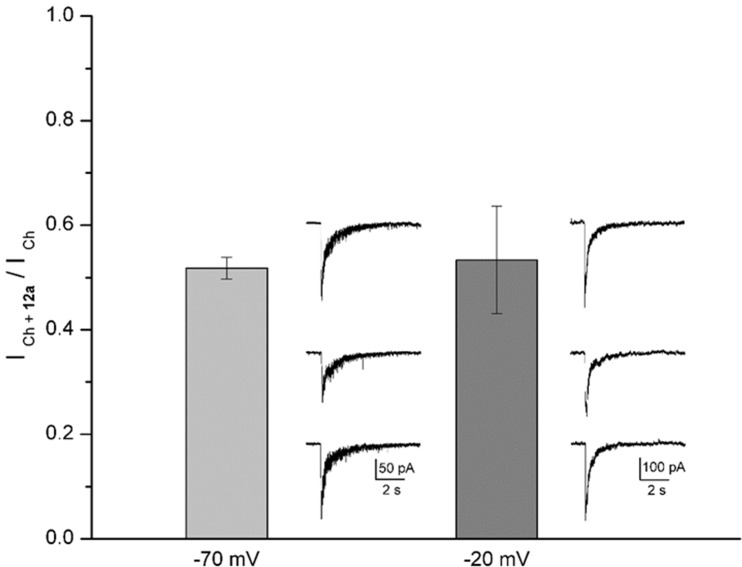
Voltage independence of **12a** effects. The height of the columns represents the ratio between the *I*_Ch_ in the presence of 5 μM **12a** (*I*_Ch__+_ **_12a_**) and the control *I*_Ch_ at the holding potential indicated. A representative control, inhibited, and recovered *I*_Ch_ (upper, middle, and bottom traces, respectively) recorded from the same interneuron are illustrated at the right of the corresponding column. Different cells were recorded for each holding potential. Each column represents the mean ± standard error with no statistical difference (*n* = 5 interneurons).

At a **12a** concentration near to that producing 50% inhibition (5 μM, see **Figure 4B**), we explored the dependence of its effects on membrane potential, which might indicate if **12a** interacts with a site located inside the ion channel or at some other site (Woodhull, 1973; García-Colunga and Miledi, 1996). In this regard, the actions of **12a** were tested by maintaining the membrane potential of hippocampal interneurons at two different values (-70 and -20 mV). The inhibition the *I*_Ch_ was independent of membrane potential, with *I*_Ch__+_ **_12a_**/*I*_Ch_ ratios of 0.52 ± 0.02 and 0.53 ± 0.10 at -70 and -20 mV, respectively (**Figure 6**), suggesting that **12a** interacts with α7 nAChRs at an external domain.

### *In Silico* Results

#### Molecular Docking

**FIGURE 7:**
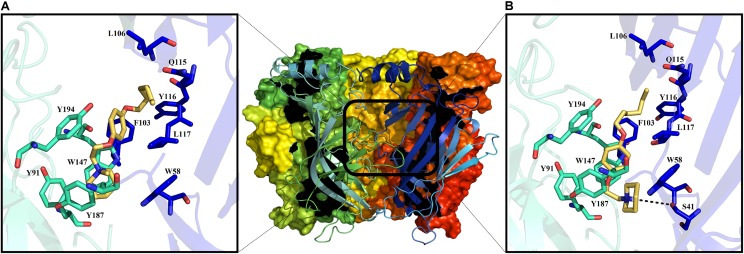
Middle: extracellular domain of the α7 nAChR. The binding site for the ligand is shown in the black rectangle. Molecular docking at the α7 nAChR active site for **12a** and **11a** **(A,B)**, respectively. Amino acid residues forming the principal (+)-side are colored in cyan, and those forming the complementary (-)-side are in blue according to the convention used for the X-ray crystal structure of *Lymnaea stagnalis* AChBP (Celie et al., 2004).

As an aid to understanding the electrophysiological results, molecular docking studies were performed for both **11a** and **12a**. The crystal structure of the rat α7 nAChR is no yet available. For this reason, a homology model was generated based on the structure of the *Aplysia californica* AChBP (**Figure 7**). It is important to mention that electrophysiological experiments were performed at pH ∼7.4. Under these conditions, the piperidine nitrogen of compound **11a** is mainly protonated.

Thus, with docking studies resulted that the quaternary ammonium group of **12a** forms cation–π interactions with the residues W147 (4.6 Å), Y91, Y187, Y191, and W58 of the principal (+)-side and the complementary (-)-side, respectively (**Figure 7A**). Additionally, the aliphatic chain of **12a**, located at the “*para*” position of the aromatic ring, presents van der Waals interactions with residues L106, Q115, Y116, and L117 of the complementary (-)-side, while the aromatic ring generates a van der Waals interaction with F103 (**Figure 7A**), which seems to confer selectivity for the α7 nAChR (Huang et al., 2006).

On the other hand, **11a** might establish a hydrogen bond with S41 of the principal (+)-side and forms π–π interactions with W147 of the complementary (-)-side; the aliphatic chain of **11a** also forms van der Waals interactions with L106 and Q115 (**Figure 7B**). The calculated binding energies for derivatives **11a** and **12a** are considerably higher than that for Ch (**Table 1**), suggesting that these compounds interact strongly with the active site of α7 nAChRs, preventing Ch from binding to the receptor, in agreement with the electrophysiological results. In this regard, the stability of their complexes might explain the slow recovery of the ability of the channel to open in response to Ch.

**Table 1:** Main interactions and binding energy of the compounds Ch, **11a** and **12a** in the α7 nAChR.

Compound	ΔG (kcal/mol)	Interaction/amino acids in the α7 nAChR
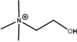
	**-3.67**	Cation–π: **(+)** **Y91**; **(+) Y187**; **(+) Y194**; **(+) W147**; **(-) W58**
**Choline**		Hydrogen bond: **(+)** **S146**
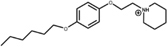
	**-8.13**	Cation–π: **(+)** **Y91**; **(+) Y187**; **(+) Y194**; **(+) W147**; **(-) W58**Hydrogen bond: **(-)** **S41**
**11a**		van der Waals: **(-)** **L106**; **(-)** **Q115**
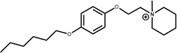
	**-8.66**	Cation–π: **(+) Y91**; **(+) Y187**; **(+) Y194**; **(+) W147**; **(-) W58**van der Waals: **(-)** **L106**;
**12a**		**(-)** **Q115**


#### Molecular Dynamics Simulations

Docking and molecular dynamic simulations are useful methodologies to obtain structural information of protein–ligand complexes. Here, the dynamics of α7/**11a** and α7/**12a** complexes were simulated during 20 ns in order to obtain information about molecular features of the systems.

##### α7/12a complex

**FIGURE 8:**
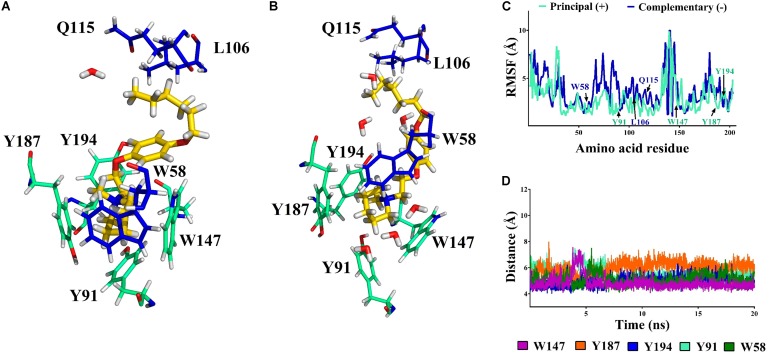
Compound **12a** (in yellow) in the binding cavity of the α7 nAChR. **(A,B)** Shows the α7/**12a** complex at 0 and 20 ns, respectively. All amino acids from the principal (+)-and complementary (-)- chains a within 5 Å are displayed (cyan and blue, respectively). **(C)** The root mean square fluctuation (RMSF) values of the residues in the principal (+)- and complementary (-)-chains show the stability of the poses of the amino acids involved at the binding site during simulation. **(D)** The distance vs. time from nitrogen atom (N_C_) of **12a** to the center of the aromatic amino acids (aromatic box) is shown.

Molecular interactions from docking studies show that **12a** interacts with amino acids Y91, Y187, Y194, W147 of the principal (+)-side and W58, F103, L106, Q115, Y116, and L117 of the complementary (-) chain of the α7 nAChR. In **Figures 8A,B** the α7/**12a** complex is displayed at 0 and 20 ns respectively, interacting with amino acid residues and water molecules. During the simulation the system was stable [root-mean-square deviation (RMSD) ∼3 Å, see Supplementary Figure 14] and the aromatic cage, characteristic of nAChRs, was conserved (**Figure 8C**). Main interactions were selected and represented in **Figure 8**.

The distances from the quaternary nitrogen atom (N_C_) of **12a** to the center of the Y187, Y194, W147, Y91, and W58 were evaluated (**Figure 8D**). The N_C_-Y187 distance (orange) shows a fluctuation associated with an interaction with a water molecule. In the case of W147 (purple) a cation–π interaction was conserved throughout almost the total simulation time. However, between 3 and 5 ns the N_C_-W147 distance increased because of a reorganization of some amino acid side chains into the cavity. After 5 ns the original distance was recovered and maintained during the remaining simulation. It is important to mention that W147 is a crucial residue for the affinity of α7 nAChR ligands. Here, the distance remains at around 4.7 Å which is in accordance with Celie et al. (2004).

##### α7/11a complex

**FIGURE 9:**
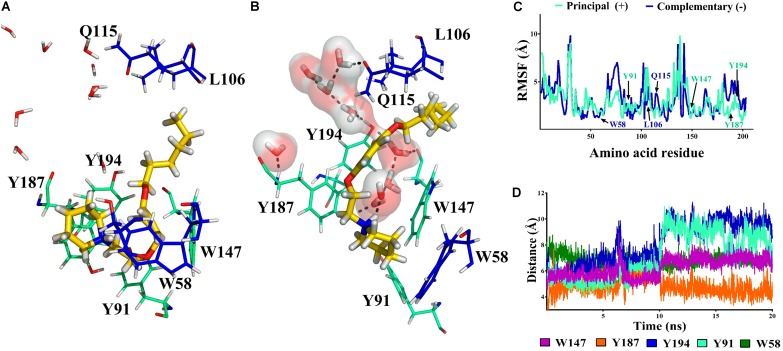
Compound **11a** (in yellow) in the binding cavity of the α7 nAChR. **(A,B)** Shows the α7/**11a** complex at 0 and 20 ns, respectively. All amino acids from the principal (+)- and complementary (-)- chains at 5 Å are displayed (cyan and blue respectively). **(C)** The RMSF values of the amino acids in the principal (+)- and complementary (-)-chains show that the position of alpha carbon (Cα) of the residues involved at the binding site persist during the simulation (W147, W58, and Y187). **(D)** The distance from the nitrogen atom (N_C_) of **11a** to the closer amino acids is shown.

The *N*-protonated ligand **11a** was also evaluated by molecular simulation in complex with α7 nAChRs. This molecule forms cation–π interactions with W58 in the receptor cavity from the beginning of the simulation. In addition, during the whole dynamics simulation the aliphatic chain of **11a** participates in van der Waals interactions with Q115 and L106 that help to stabilize the ligand in the cavity (**Figure 9A**).

A short hydrogen bond (1.9 Å) between the Y187 (Schiøtt et al., 1998) and the –NH of **11a** was observed at 10 ns due to the entry of a water molecule into the binding cavity. Consequently, the interaction with Y187 was broken and the cavity was solvated. Thus, W147, Y187, and Y194 from the principal (+)-chain and Q115 from the complementary (-)-chain generate a network of hydrogen bonds which persists during all the simulation (**Figure 9B**). Therefore, based on the cavity solvation at 10 ns the distance between the aromatic ring of Y194, Y91 and the nitrogen of **11a** increase by more than 5 Å (**Figure 9D**) but no effect was observed on the amino acids W147, W58, and Y187 (**Figure 9C**).

##### Role of water molecules

Our atomistic simulations of **11a** and **12a** interacting with α7 nAChRs show an interesting difference in the main interactions inside the cavity associated with a solvation network. In this context, a potential role of water molecules could be related to the decrease of the antagonistic effect in the derivatives studied here.

**FIGURE 10:**
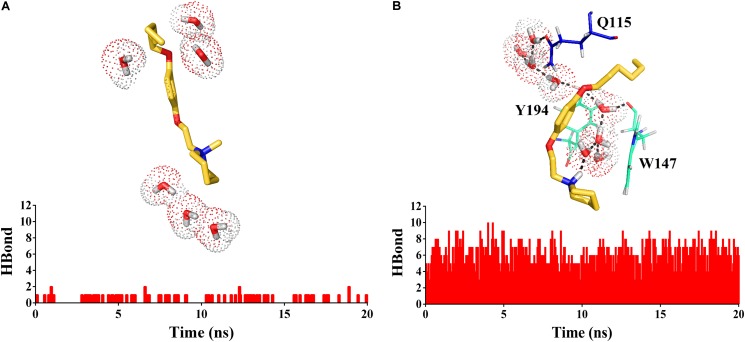
Histogram of hydrogen bonds during the molecular dynamics simulation for **12a** **(A)** and **11a** **(B)**. In **(B)** the hydrogen bonds network is represented with dashed lines.

The 1-[2-(4-alkyloxy-phenoxy-ethyl)]-1-methylpiperidinium iodide derivatives (**12a**-**12c**) are potent antagonists of α7 nAChRs, governed by a cation–π interaction in the receptor’s aromatic box. This result is consistent with the stabilization of the receptor in a non-active state. On the other hand, the 1-[2-(4-alkyloxy-phenoxy-ethyl)]piperidine derivatives (**11a**-**11c**) have a hydrogen bond donator (-NH) which allows a hydrogen bond to form that interacts with Q115, W147 and Y194, generating as a consequence a more flexible cavity (**Figures 10A,B**).

## Discussion

In the present work, chemical synthesis, pharmacological activity, molecular docking studies, and molecular dynamics simulations of a new series of 1-[2-(4-alkyloxy-phenoxy-ethyl)]piperidines (**11a**-**11c**) and 1-[2-(4-alkyloxy-phenoxy-ethyl)]-1-methylpiperidinium iodides (**12a**-**12c**) were performed to evaluate them as possible antagonists of α7 nAChRs (see **Figure 3**).

**FIGURE 11:**
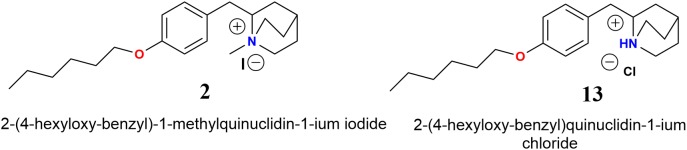
Molecular structures of *N*-methylquinolidinium iodides **2** and quinuclidinium chloride **13** (Arias et al., 2013).

Electrophysiological recordings of the *I*_Ch_ in interneurons from the *stratum radiatum* hippocampal CA1 area indicated that compounds **12a**-**12c** inhibited the *I*_Ch_ more strongly than the corresponding **11a**-**11c**, **12a** showing the most potent antagonistic effect on α7 nAChRs. Furthermore, we found that alkyl carbon chains of five atoms led to weaker *I*_Ch_ inhibition than compound **11a** (**Figure 4**). These results agree with previous reports, where compounds that present alkylated nitrogen incorporating an aromatic ring substituted in the “*para*” position with aliphatic chains containing six atoms of carbon are more potent antagonists for α7 nAChRs than those with non-alkylated nitrogen (compounds **2** and **13**, respectively, **Figure 11**; Arias et al., 2013).

The fact that interneurons from the *stratum radiatum* hippocampal CA1 area express α7-containing nAChRs including homomeric α7 and heteromeric α7β2 nAChRs (Wu et al., 2016), it is very likely that the *I*_Ch_ is the mixture of responses mediated by both subtypes of receptors. Thus, one possibility is that **12a** inhibited both α7 and α7β2 nAChRs. In this regard, the compound **12a** or some of its derivatives may be useful for the treatment of some diseases such as major depression and/or different types of cancer, in which α7 nAChRs antagonists reverted these conditions (Egleton et al., 2008; Mineur et al., 2017).

The molecular docking studies and molecular dynamics simulations help to explain electrophysiological results. In this regard, **12a** forms cation–π interactions with the aromatic cage of the α7 nAChR, important for ligand affinity to the α7 nAChR (Celie et al., 2004). In addition, the aliphatic chain of **12a** presents van der Waals interactions with F103, L106, Q115, Y116, and L117 of the complementary (-)-side. On the other hand, molecular dynamics studies showed that the *N*-methyl group of **12a** and Y194 establish a van der Waals interaction. All these interactions were conserved during almost all the molecular dynamics simulation time (20 ns), preventing both conformational changes of the receptor and its activation, which may account for the slow recovery of the *I*_Ch_ inhibition observed in electrophysiological assays (**Figure 8** and Supplementary Figure 15). The *N*-methyl group on the piperidinium segment confers a positive charge in the nitrogen atom (N_C_), crucial for a cation–π interaction with the aromatic residues into the binding cavity (high electron density aromatic box; Zhong et al., 1998). This is the same case of methylation on N_C_ for the nicotine pyrrolidone ring and of carbamylcholine (Celie et al., 2004).

To establish whether piperidine derivatives could interact with the heteromeric rat α9α10 nAChR, a docking study was performed for the compound **12a** at the active site of this receptor [interface between α10(+) and α9(-); Yu et al., 2013; Azam et al., 2015]. For this purpose, we used the structure of the human α4β2 nAChR (Morales-Perez et al., 2016). In this regard, these studies suggest that the quaternary ammonium group of **12a** also generates cation–π interactions with the aromatic box (Y91, Y187, Y194, W147, and W58) of the α9α10 nAChR. Interestingly and in contrast with the α7 nAChR (see Supplementary Figure 16A), the aliphatic chain and the aromatic ring of **12a** do not generate van der Waals interactions with the residues of the complementary (-)-side chain of the α9α10 nAChR, due to the presence of D117, which generates a repulsive effect by its negative charge, causing the aliphatic chain and the aromatic ring to move away from this area. Furthermore, the aliphatic chain of **12a** presents only weak van der Waals interactions with A130 and L131 (see Supplementary Figure 16B).

These results indicate that the compound **12a** could interact at the binding site of the α9α10 nAChR; however with less binding energy. This may be explained because the complementary (-)-side of the α9 subunit would be very different from the complementary (-)-side of the α7 subunit, which may be important for selectivity of **12a** for the α7 nAChR (see Supplementary Figure 16C).

Regarding the non-methylated compound **11a**, the piperidine nitrogen of the compound is protonated at physiological pH, producing a hydrogen bond that forms a solvation network with the water molecules in the binding cavity of the α7 nAChR. As in the case of **12a**, during the molecular dynamics simulations the aliphatic chain of **11a** maintains van der Waals interactions with Q115, helping to stabilize the ligand in the cavity. Thus, the difference in the interaction of **12a** and **11a** with the α7 nAChR is the cation–π interactions and hydrogen bonds, respectively, may account for the diminution of the antagonist activity, and then less *I*_Ch_ inhibition by **11a**. For instance, ligands with groups forming hydrogen bonds that are able to generate a solvation network with the water molecules and bind with Q115 are selective agonists of the α7 nAChR (Huang et al., 2006). Consequently, one possibility is that **11a** may be, in addition to a slight antagonist, a partial agonist of the α7 nAChR. However, 10 μM **11a** applied alone both in the bath solution or with pressure puffs had no measurable effect on the membrane ion current (data not shown, *n* = 3 for each condition), discarding an agonist action on α7 nAChR.

Inhibition of the *I*_Ch_ by **12a**, the most potent of these antagonists of α7 nAChRs, was independent of membrane potential. Moreover, the fact that complete inhibition of the *I*_Ch_ by **12a** occurred between 1 and 10 μM, resulting in a sharp slope of the concentration-response relation, may indicate a high degree of cooperativity. All these suggest several binding sites for **12a** possibly located in an external domain of the receptor, similarly as the interactions of methyllycaconitine and imipramine at chick and human α7 nAChRs, respectively (Palma et al., 1996; Arias et al., 2018). These results are consistent with the present molecular docking studies and molecular dynamics simulation. Other substances interact with nAChRs in multiple sites, exhibiting complex functional modulation of the receptor; this is the case of Zn^2+^, La^3+^, atropine, tacrine and the compound 2 (García-Colunga and Miledi, 1997; Zwart and Vijverberg, 1997; Moroni et al., 2008; López et al., 2015). In the case of (α4)_3_(β2)_2_ nAChRs, there are the three interacting sites for Zn^2+^ (two for inhibition and one for potentiation) (García-Colunga and Miledi, 1997; Zwart and Vijverberg, 1997; Moroni et al., 2008). Furthermore, compound **2** interacts with at least two sites in the ion channel/receptor complex: one for potentiating and another for inhibiting the α7 nAChRs (López et al., 2015).

## Conclusion

The main purpose of this study was the designing, synthesis, and chemical characterization of a new series of 1-[2-(4-alkoxy-phenoxy-ethyl)]piperidines and 1-[2-(4-alkyloxy-phenoxy-ethyl)]-1-methylpiperidinium iodides, as well as their biological and computational appraisal for evaluating them as potential ligands for the α7 nAChR. With the results presented here we propose that the mode of interaction of **12a** is better than that of **11a** for having antagonist effects on the α7 nAChR in a competitive fashion. *In silico* studies indicated that **12a** generated cation–π and van der Waals interactions which were preserved throughout the simulation time. While **11a** by presenting a hydrogen bridge-forming (NH) group, generated hydrogen bonds with water molecules within the cavity, preventing the formation of a cation–π interaction. Water molecules inside the receptor cavity would play an important role in decreasing antagonistic activity in non-methylated derivatives, due to the formation of hydrogen bonds, which would lead to a more flexible cavity. We hope that this study may lead to development of new compounds with antagonistic properties at α7 nAChRs that might be clinically useful for the treatment of pathologies associated with these receptors, including neuropsychiatric disorders and different types of cancer.

## Author Contributions

JJL and JG-C: performed the research and analyzed the data. JG-C, EP, and AF supervised the investigation. All the authors designed the research, wrote, and critically revised the manuscript.

## Conflict of Interest Statement

The authors declare that the research was conducted in the absence of any commercial or financial relationships that could be construed as a potential conflict of interest.

## References

[B1] Albuquerque E. X. Pereira E. F. R. Alkondon M. Rogers S. W. (2009). Mammalian nicotinic acetylcholine receptors: from structure to function. *Physiol. Rev.* 89 73–120. 10.1152/physrev.00015.2008 19126755 PMC2713585

[B2] Alkondon M. Pereira E. Cortes W. Maelicke A. Albuquerque E. (1997). Choline is a selective agonist of α7 nicotinic acetylcholine receptors in the rat brain neurons. *Eur. J. Neurosci.* 9 2734–2742. 10.1111/j.1460-9568.1997.tb01702.x 9517478

[B3] Arias H. R. López J. J. Feuerbach D. Fierro A. Ortells M. O. Pérez E. G. (2013). Novel 2-(substituted benzyl)quinuclidines inhibit human α7 and α4β2 nicotinic receptors by different mechanisms. *Int. J. Biochem. Cell Biol.* 45 2420–2430. 10.1016/j.biocel.2013.08.003 23954208

[B4] Arias H. R. Vázquez-Gómez E. Hernández-Abrego A. Gallino S. Feuerbach D. Ortells M. O. (2018). Tricyclic antidepressants inhibit hippocampal α7^∗^ and α9α10 nicotinic acetylcholine receptors by different mechanisms. *Int. J. Biochem. Cell Biol.* 25 1–10. 10.1016/j.biocel.2018.04.017 29704625

[B5] Azam L. Papakyriakou A. Zouridakis M. Giastas P. Tzartos S. J. McIntosh J. M. (2015). Molecular interaction of α-conotoxin RgIA with the rat α9α10 nicotinic acetylcholine receptor. *Mol. Pharmacol.* 87 855–864. 10.1124/mol.114.096511 25740413 PMC4407738

[B6] Blum A. P. Lester H. A. Dougherty D. A. (2010). Nicotinic pharmacophore: the pyridine *N* of nicotine and carbonyl of acetylcholine hydrogen bond across a subunit interface to a backbone NH. *Proc. Natl. Acad. Sci. U.S.A.* 107 13206–13211. 10.1073/pnas.1007140107 20616056 PMC2922130

[B7] Celie P. H. N. van Rossum-Fikkert S. E. van DijK W. J. Brejc K. Smit A. B. Sixma T. K. (2004). Nicotine and carbamylcholine binding to nicotinic acetylcholine receptors as studied in AChBP crystal structures. *Neuron* 41 907–914. 10.1016/S0896-6273(04)00115-1 15046723

[B8] Cooke J. P. Bitterman H. (2004). Nicotine and angiogenesis: a new paradigm for tobacco-related diseases. *Ann. Med.* 36 33–40. 10.1080/07853890310017576 15000345

[B9] Corpet F. (1988). Multiple sequence alignment with hierarchical clustering. *Nucleic Acids Res.* 16 10881–10890. 10.1093/nar/16.22.10881 2849754 PMC338945

[B10] Crooks P. A. Ravard A. Wilkins L. H. Teng L. H. Buxton S. T. Dwoskin L. P. (1995). Inhibition of nicotine-evoked [3H]dopamine release by pyridino N-substituted nicotine analogs: a new class of nicotinic antagonist. *Drug Dev. Res.* 36 91–102. 10.1002/ddr.430360204

[B11] Dang N. Meng X. Song H. (2016). Nicotinic acetylcholine receptors and cancer. *Biomed. Rep.* 4 515–518. 10.3892/br.2016.625 27123240 PMC4840641

[B12] Dasgupta P. Rastogi S. Pillai S. Ordonez-Ercan D. Morris M. Haura E. (2006). Nicotine induces cell proliferation by β-arrestin-mediated activation of Src and Rb–Raf-1 pathways. *J. Clin. Invest.* 116 2208–2217. 10.1172/JCI28164 16862215 PMC1513051

[B13] Dineley K. T. Pandya A. A. Yakel J. L. (2015). Nicotinic ACh receptors as therapeutic targets in CNS disorders. *Trends Pharmacol. Sci.* 36 96–108. 10.1016/j.tips.2014.12.002 25639674 PMC4324614

[B14] Dunbar G. C. Kuchibhatla R. V. Lee G. (2011). A randomized double-blind study comparing 25 and 50 mg TC-1734 (AZD3480) with placebo, in older subjects with age-associated memory impairment. *J. Psychopharmacol.* 25 1020–1029. 10.1177/0269881110367727 20542923

[B15] Dwoskin L. P. Crooks P. A. (2001). Competitive neuronal nicotinic receptor antagonists: a new direction for drug discovery. *J. Pharmacol. Exp. Ther.* 298 395–402. 10.1016/j.bcp.2013.07.021 11454899

[B16] Egleton R. D. Brown K. C. Dasgupta P. (2008). Nicotinic acetylcholine receptors in cancer: multiple roles in proliferation and inhibition of apoptosis. *Trends Pharmacol. Sci.* 29 151–158. 10.1016/j.tips.2007.12.006 18262664

[B17] Frisch M. J. Trucks G. W. Schlegel H. B. Scuseria G. E. Robb M. A. Cheeseman J. R. (2004). Gaussian 03, revision C.02. *J. Comput. Chem.* 24 1748–1757.

[B18] Gahring L. C. Myers E. J. Dunn D. M. Weiss R. B. Rogers S. W. (2017). Nicotinic alpha 7 receptor expression and modulation of the lung epithelial response to lipopolysaccharide. *PLoS One* 12:e0175367. 10.1371/journal.pone.0175367 28384302 PMC5383308

[B19] García-Colunga J. Miledi R. (1996). Serotonergic modulation of muscle acetylcholine receptors of different subunit composition. *Proc. Natl. Acad. Sci. U.S.A.* 93 3990–3994. 10.1073/pnas.93.9.3990 8633003 PMC39473

[B20] García-Colunga J. Miledi R. (1997). Opposite effects of lanthanum on different types of nicotinic acetylcholine receptors. *Neuroreport* 8 3293–3296. 10.1097/00001756-199710200-00020 9351659

[B21] Ghiron C. Haydar S. N. Aschmies S. Bothmann H. Castaldo C. Cocconcelli G. (2010). Novel α7 nicotinic acetylcholine receptor agonists containing a urea moiety: identification and characterization of the potent, selective, and orally efficacious agonist 1-[6-(4-fluorophenyl)pyridin-3-yl]-3-(4-piperidin-1-ylbutyl) urea (SEN34625/WYE-103914). *J. Med. Chem.* 53 4379–4389. 10.1021/jm901692q 20465311

[B22] Gotti C. Clementi F. Fornari A. Gaimarri A. Guiducci S. Manfredi I. (2009). Structural and functional diversity of native brain neuronal nicotinic receptors. *Biochem. Pharmacol.* 78 703–711. 10.1016/j.bcp.2009.05.024 19481063

[B23] Grimster N. P. Stump B. Fotsing J. R. Weide T. Talley T. T. Yamauchi J. G. (2012). Generation of candidate ligands for nicotinic acetylcholine receptors via in situ click chemistry with a soluble acetylcholine binding protein template. *J. Am. Chem. Soc.* 134 6732–6740. 10.1021/ja3001858 22394239 PMC3618991

[B24] Heeschen C. Weis M. Aicher A. Dimmeler S. Cooke J. P. (2002). A novel angiogenic pathway mediated by nonneuronal nicotinic acetylcholine receptors. *J. Clin. Invest.* 110 527–536. 10.1172/JCI200214676 12189247 PMC150415

[B25] Huang X. Zheng F. Chen X. Crooks P. A. Dwoskin L. P. Zhan C. G. (2006). Modeling subtype-selective agonists binding with α4β2 and α7 nicotinic acetylcholine receptors: effects of local binding and long-range electrostatic interactions. *J. Med. Chem.* 49 7661–7674. 10.1021/jm0606701 17181149

[B26] Innocent N. Livingstone P. D. Hone A. Kimura A. Young T. Whiteaker P. (2008). αConotoxin Arenatus IB[V11L,V16D] Is a potent and selective antagonist at rat and human native α7 nicotinic acetylcholine receptors. *J. Pharmacol. Exp. Ther.* 327 529–537. 10.1124/jpet.108.142943 18664588 PMC2596936

[B27] Lester H. A. Dibas M. I. Dahan D. S. Leite J. F. Dougherty D. A. (2004). Cys-loop receptors: new twists and turns. *Trends Neurosci.* 27 329–336. 10.1016/j.tins.2004.04.002 15165737

[B28] Liu Q. Huang Y. Shen J. Steffensen S. Wu J. (2012). Functional α7β2 nicotinic acetylcholine receptors expressed in hippocampal interneurons exhibit high sensitivity to pathological level of amyloid β peptides. *BMC Neurosci.* 13:155. 10.1186/1471-2202-13-155 23272676 PMC3573893

[B29] López J. J. Pérez E. G. García-Colunga J. (2015). Dual effects of a 2-benzylquinuclidinium derivative on α7-containing nicotinic acetylcholine receptors in rat hippocampal interneurons. *Neurosci. Lett.* 607 35–39. 10.1016/j.neulet.2015.09.016 26384784

[B30] Mazurov A. A. Speake J. D. Yohannes D. (2011). Discovery and development of α7 nicotinic acetylcholine receptor modulators. *J. Med. Chem.* 54 7943–7961. 10.1021/jm2007672 21919481

[B31] Mineur Y. S. Mose T. N. Blakeman S. Picciotto M. R. (2017). Hippocampal α7 nicotinic ACh receptors contribute to modulation of depression-like behaviour in C57BL/6J mice. *Br. J. Pharmacol.* 175 1903–1914. 10.1111/bph.13769 28264149 PMC5979617

[B32] Morales-Perez C. L. Noviello C. M. Hibbs R. E. (2016). X-ray structure of the human α4β2 nicotinic receptor. *Nature* 538 411–415. 10.1038/nature19785 27698419 PMC5161573

[B33] Moroni M. Vijayan R. Carbone A. Zwart R. Biggin P. C. Bermudez I. (2008). Non-agonist-binding subunit interfaces confer distinct functional signatures to the alternate stoichiometries of the α4β2 nicotinic receptor: an α4-α4 interface is required for Zn2 + potentiation. *J. Neurosci.* 28 6884–6894. 10.1523/JNEUROSCI.1228-08.2008 18596163 PMC3844799

[B34] Morris G. M. Goodsell D. S. Halliday R. S. Huey R. Hart W. E. Belew R. K. (1998). Automated docking using a Lamarckian genetic algorithm and empirical binding free energy function. *J. Comput. Chem.* 19 1639–1662. 10.1002/(SICI)1096-987X(19981115)19:14<1639::AID-JCC10>3.0.CO;2-B

[B35] Olsen J. A. Balle T. Gajhede M. Ahring P. K. Kastrup J. S. (2014). Molecular recognition of neurotransmitter acetylcholine by an acetylcholine binding protein reveals determinants of binding to nicotinic acetylcholine receptors. *PLoS One* 9:e91232. 10.1371/journal.pone.0091232 24637639 PMC3956608

[B36] Palma E. Bertrand S. Binzoni T. Bertrand D. (1996). Neuronal nicotinic α7 receptor expressed in *Xenopus* oocytes presents five putative binding sites for methyllycaconitine. *J. Physiol.* 491 151–161. 10.1113/jphysiol.1996.sp021203 9011607 PMC1158766

[B37] Peng Y. Zhang Q. Snyder G. L. Zhu H. Yao W. Tomesch J. (2010). Discovery of novel α7 nicotinic receptor antagonists. *Bioorg. Med. Chem. Lett.* 20 4825–4830. 10.1016/j.bmcl.2010.06.103 20638843 PMC3000800

[B38] Pérez E. G. Cassels B. K. Eibl C. Gündisch D. (2012). Synthesis and evaluation of *N*1-alkylindole-3-ylalkylammonium compounds as nicotinic acetylcholine receptor ligands. *Bioorg. Med. Chem. Lett.* 20 3719–3727. 10.1016/j.bmc.2012.04.050 22609074

[B39] Pérez E. G. Ocampo C. Feuerbach D. López J. J. Morelo G. L. Tapia R. A. (2013). Novel 1-(1-benzyl-1H-indol-3-yl)-*N,N,N*-trimethylmethanaminium iodides are competitive antagonists for the human α4β2 and α7 nicotinic acetylcholine receptors. *Med. Chem. Commun.* 4 1166–1170. 10.1039/c3md00042g

[B40] Peters D. Eriksen B. L. Nielsen E. O. Scheel-Krüger J. Olsen G. M. (2006). inventor; Neurosearch A/S., assignee. Novel 8-aza-bicyclo[3.2.1]octane derivatives and their use as monoamine neurotransmitter re-uptake inhibitors. United States Patent WO2004072075 A1.

[B41] Phillips J. C. Braun R. Wang W. Gumbart J. Tajkhorshid E. Villa E. (2005). Scalable molecular dynamics with NAMD. *J. Comput. Chem.* 26 1781–1802. 10.1002/jcc.20289 16222654 PMC2486339

[B42] Romanelli M. N. Gratteri P. Guandalini L. Martini E. Bonaccini C. Gualtieri F. (2007). Central nicotinic receptors: structure, function, ligands, and therapeutic potential. *Chem. Med. Chem.* 2 746–767. 10.1002/cmdc.200600207 17295372

[B43] Roy A. Reddy K. R. Mohanta P. K. Ila H. Junjappat H. (1999). Hydrogen peroxide/boric acid: an efficient system for oxidation of aromatic aldehydes and ketones to phenols. *Synth. Commun.* 29 3781–3791. 10.1080/00397919908086017

[B44] Šali A. Blundell T. L. (1993). Comparative protein modelling by satisfaction of spatial restraints. *J. Mol. Biol.* 234 779–815. 10.1006/jmbi.1993.1626 8254673

[B45] Schiøtt B. Iversen B. B. Madsen G. K. Larsen F. K. Bruice T. C. (1998). On the electronic nature of low-barrier hydrogen bonds in enzymatic reactions. *Proc. Natl. Acad. Sci. U.S.A.* 95 12799–12802. 10.1073/pnas.95.22.12799 9788994 PMC23598

[B46] Terry A. V. Jr. Callahan P. M. Hernandez C. M. (2015). Nicotinic ligands as multifunctional agents for the treatment of neuropsychiatric disorders. *Biochem. Pharmacol.* 97 388–398. 10.1016/j.bcp.2015.07.027 26231940 PMC4752163

[B47] Trombino S. Cesario A. Margaritora S. Granone P. Motta G. Falugi C. (2004). α7-Nicotinic acetylcholine receptors affect growth regulation of human mesothelioma cells: role of mitogen activated protein kinase pathway. *Cancer Res.* 64 135–145. 10.1158/0008-5472.CAN-03-1672 14729617

[B48] Vanommeslaeghe K. Hatcher E. Acharya C. Kundu S. Zhong S. Shim J. (2010). CHARMM General force field: a force field for drug-like molecules compatible with the CHARMM all-atom additive biological force field. *J. Comput. Chem.* 31 671–690. 10.1002/jcc.21367 19575467 PMC2888302

[B49] Vázquez-Gómez E. Arias H. R. Feuerbach D. Miranda-Morales M. Mihailescu S. Targowska-Duda K. M. (2014). Bupropion-induced inhibition of α7 nicotinic acetylcholine receptors expressed in heterologous cells and neurons from dorsal raphe nucleus and hippocampus. *Eur. J. Pharmacol.* 740 103–111. 10.1016/j.ejphar.2014.06.059 25016090

[B50] Wilkins L. H. Grinevich V. P. Ayers J. T. Crooks P. A. Dwoskin L. P. (2002). *N*-n-alkylnicotinium analogs, a novel class of nicotinic receptor antagonists: interaction with α4β2^∗^ and α7^∗^ neuronal nicotinic neceptors. *J. Pharmacol. Exp. Ther.* 304 400–410. 10.1124/jpet.102.043349 12490617

[B51] Wong H. P. Yu L. Lam E. K. Y. Tai E. K. K. Wu W. K. K. Cho C. H. (2007). Nicotine promotes cell proliferation via α7-nicotinic acetylcholine receptor and catecholamine-synthesizing enzymes-mediated pathway in human colon adenocarcinoma HT-29 cells. *Toxicol. Appl. Pharmacol.* 221 261–267. 10.1016/j.taap.2007.04.002 17498763

[B52] Woodhull A. M. (1973). Ionic blockage of sodium channels in nerve. *J. Gen. Physiol.* 61 687–708. 10.1085/jgp.61.6.687 4541078 PMC2203489

[B53] Wu J. Liu Q. Tang P. Mikkelsen J. D. Shen J. Whiteaker P. (2016). Heteromeric α7β2 nicotinic acetylcholine receptors in the brain. *Trends Pharmacol. Sci.* 37 562–574. 10.1016/j.tips.2016.03.005 27179601 PMC5074342

[B54] Yu R. Kompella S. N. Adams D. J. Craik D. J. Kaas Q. (2013). Determination of the α-conotoxin Vc1.1 binding site on the α9α10 nicotinic acetylcholine receptor. *J. Med. Chem.* 56 3557–3567. 10.1021/jm400041h 23566299

[B55] Zhong W. Gallivan J. P. Zhang Y. Li L. Lester H. A. Dougherty D. A. (1998). From ab initio quantum mechanics to molecular neurobiology: a cation-π binding site in the nicotinic receptor. *Proc. Natl. Acad. Sci. U.S.A.* 95 12088–12093. 10.1073/pnas.95.21.12088 9770444 PMC22789

[B56] Zoli M. Pucci S. Vilella A. Gotti C. (2017). Neuronal and extraneuronal nicotinic acetylcholine receptors. *Curr. Neuropharmacol.* 15 1–11. 10.2174/1570159X15666170912110450 28901280 PMC6018187

[B57] Zwart R. Vijverberg H. P. (1997). Potentiation and inhibition of neuronal nicotinic receptors by atropine: competitive and noncompetitive effects. *Mol. Pharmacol.* 52 886–895. 10.1124/mol.52.5.886 9351980

