# Supplementary material for: Methylpiperidinium Iodides as Novel Antagonists for α7 Nicotinic Acetylcholine Receptors
Source: Front Pharmacol. 2018 Jul 10;9:744. doi: 10.3389/fphar.2018.00744 (PMC6048275; doi:10.3389/fphar.2018.00744)
Supplement: Supplementary file 1 [file Data_Sheet_1.DOCX]

Supplementary Material

Article Title

**Jhon Jairo López*, Jesús García-Colunga*, Edwin Pérez, Angélica Fierro**

**Corresponding Author**: Jhon Jairo López, jjlopez@uc.cl or Jesús García-Colunga, garciacolunga@unam.mx

# Supplementary Data

**Experimental Section**

Reagents and solvents: 4-Hydroxybenzaldehyde, iodomethane (CH_3_I), 1-bromohexane, 1-bromoheptane, 1-bromooctane, 2-piperidineethanol, diisopropyl azodicarboxylate (DIAD), triphenylphosphine (PPh_3_), potassium carbonate (K_2_CO_3_), 10% sodium bicarbonate (NaHCO_3_), sodium borohydride (NaBH_4_), anhydrous sodium sulfate (Na_2_SO_4_), boric acid (H_3_BO_3_), 30% hydrogen peroxide (H_2_O_2_), sulfuric acid (H_2_SO_4_), aqueous ammonia, Celite 545 (0.02-0.1 mm; MERCK), methanol, ethanol, acetone, dichloromethane, diethyl ether, hexane, ethyl acetate, tetrahydrofuran (THF) and 1,4-dioxane.

**General**

All solvents and all deuterated solvents were purchased from Merck, reagents from Aldrich, Merck and AK Scientific. Column chromatography was performed with silica gel (Merck, type 60, 0.063-0.2 mm). Nuclear Magnetic Resonance (NMR) spectra were recorded on a Bruker Avance 400 MHz spectrometer. All chemical shifts in NMR experiments were reported in δ values (parts per million, ppm) downfield from tetramethyl-silane. Melting points were determined on a Reichert Galen III hot plate microscope apparatus and are non-corrected.

**General procedure for preparation of substituted benzaldehydes**

The Williamson method was initially used to couple 4-hydroxylbenzaldehyde (**8**) and alkyl halides to produce the corre-sponding alkyloxy benzaldehydes. In round-bottomed flask 100 mL fitted with a magnetic stir bar is charged with K_2_CO_3_ (6 g, 43.4 mmol), ethanol (30 mL), 4-hydroxylbenzaldehyde (6 g, 49.1 mmol) and alkyl halides (4.0 mL, 49.1 mmol). The mixture was refluxed for 18 hours under nitrogen with stirring. The resulting suspension was filtered under vacuum and the solvent removed under reduced pressure on a rotary evaporator. Finally, the compound was purified by column chromatography (silica gel from 0.063 to 0.2 mm, CH_2_Cl_2_) to affording the substituted benzaldehydes

**General procedure for preparation of substituted phenols**

In a round bottom flask of 100 mL were added 30 mL THF, H_3_BO_4_ (3.1 g, 50.13 mmol), H_2_O_2_ (2.5 g, 30% in water, 73.52 mmol) and 1 mL of H_2_SO_4_. The mixture was stirring for 0.5 hours at room temperature. In another round bottom flask of 100 mL were dissolved the substituted benzaldehydes (9.99 mmol) in 10 mL of THF. This mixture was added drop by drop the first ball (H_3_BO_3_-H_2_O_2_-THF-H_2_SO_4_). Then the reaction mixture was allowed to stir for 24 hours at room temperature. After this time, the reaction mixture was filtered and washed with THF. Subsequently filtering was neutralized with NaHCO_3_ solution at 10% and extracted with chloroform (3 x 25 mL). The layer organic was washed with water (50 mL) dried over anhydrous Na_2_SO_4_ and concentrated under reduced pressure. The product was purified by chromatography column (from 0.063 to 0.2 mm silica gel, Hexane-ethyl acetate 7:3) to affording the substituted phenols (**10a-10c**).

**General procedure preparation of the derivatives of 1-[2-(4-alkyloxy-phenoxy-ethyl)] piperidine and 1-[2-(4-alkyloxy-phenoxy-ethyl)]-1-methylpiperidinium iodides**

In a flat flask with two mouths was added the substituted phenols (**10a-10c**; 2.56 mmol), 2-piperidineethanol (283 μL, 2.13 mmol) and 15 mL of 1,4-dioxane. The mixture was allowed to stir under nitrogen and subsequently PPh_3_ is added (840 mg, 3.2 mmol). The reaction was cooled to 0 °C and and then the DIAD (406 μL, 3.2 mmol in 10 mL of 1,4-dioxane) solution is slowly added. The mixture was refluxed for 48 hours under nitrogen with stirring. After this time, the reaction mixture was concentrated under reduced pressure and the product was purified by chromatography column (silica gel from 0.063 to 0.2 mm Dichloromethane-Methanol-Aqueous ammonia 9:1:0.5) to affording the derivatives of 1-[2-(4-alkyloxy-phenoxy-ethyl)] piperidine (**11a-11c**). Subsequently these compounds (0.34 mmol) were dissolved in 2 mL of acetone. Methyl iodide is added and then the reaction mixture was allowed to stir for 16 hours at room temperature. Ethyl ether is then added (10 mL) and a viscous precipitate is formed. The viscous liquid is then separated by filtration, washing with ethyl ether (3 x 30 mL) and concentrate under reduced pressure to affording the derivatives 1-[2-(4-alkyloxy-phenoxy-ethyl)]-1-methylpiperidinium iodides (**12a-12c**).

# Supplementary Figures and Tables

**Analytical data of New Compounds**

**1-[2-(4-(Hexyloxy)phenoxy)ethyl]piperidine (11a)**

Orange liquid; ^1^H NMR (400 MHz, CDCl_3_) δ 6.81 (m, 4H), 4.04 (t, *J* = 6.1 Hz, 2H), 3.88 (t, *J* =6.6 Hz, 2H), 2.74 (t, *J* = 6.1 Hz, 2H), 2.50 (s, 2H), 1.74 (m, 4H), 1.60 (m, 4H), 1.43 (m, 4H), 1.32 (dd, *J* = 8.7, 5.2 Hz, 4H), 0.90 (t, *J* = 6.6 Hz, 3H). ^13^C NMR (101 MHz, CDCl_3_) δ 153.36, 152.86, 115.47, 115.33, 68.56, 66.49, 58.02, 54.99, 31.59, 29.34, 25.79, 25.72, 24.17, 22.59, 14.01.

**1-[2-(4-(Heptyloxy)phenoxy)ethyl]piperidine (11b)**

Brown liquid; ^1^H NMR (400 MHz, CDCl_3_) δ 6.81 (m, 4H), 4.12 (t, *J* = 5.7 Hz, 2H), 3.88 (t, *J* = 6.6 Hz, 2H), 2.87 (t, *J* = 5.7 Hz, 2H), 2.64 (s, 2H), 1.70 (m, 6H), 1.45 (m, 4H), 1.32 (m, 6H), 0.89 (t, *J* = 6.7 Hz, 3H). ^13^C NMR (101 MHz, CDCl_3_) 153.52, 152.53, 115.49, 115.38, 68.58, 65.88, 57.69, 54.78, 31.77, 29.36, 29.05, 26.00, 25.25, 23.70, 22.58, 14.06.

**1-[2-(4-(Octyloxy)phenoxy)ethyl]piperidine (11c)**

Orange liquid; ^1^H NMR (400 MHz, CDCl_3_) δ 6.81 (m, 4H), 4.13 (t, *J* = 5.5 Hz, 2H), 3.88 (t, *J* = 6.5 Hz, 2H), 2.89 (t, *J* = 5.5 Hz, 2H), 2.67 (s, 2H), 1.72 (m, 6H), 1.46 (m, 4H), 1.28 (m, 8H), 0.87 (d, *J* = 6.8 Hz, 3H). ^13^C NMR (101 MHz, CDCl_3_) δ 153.55, 152.49, 115.51, 115.39, 68.60, 65.80, 57.65, 54.76, 31.79, 29.29, 29.12, 26.04, 25.17, 23.63, 22.63, 14.08.

**1-[2-(4-(heptyloxy)phenoxy)ethyl]-1-methylpiperidin-1-ium iodide (12a)**

White solid; mp: 90-94 °C; ^1^H NMR (400 MHz, CDCl_3_) δ 6.84 (m, 4H), 4.44 (d, *J* = 3.9 Hz, 2H), 4.23 (d, *J* = 3.9 Hz, 2H), 3.88 (t, *J* = 6.5 Hz, 2H), 3.80 (m, 4H), 3.48 (s, 3H), 1.99 (s, 4H),1.79 (ddd, *J* = 21.6, 21.2, 15.0 Hz, 4H), 1.44 (m, 2H), 1.34 (m, 4H), 0.90 (t, *J* = 6.2 Hz, 3H). ^13^C NMR (101 MHz, CDCl_3_) δ 154.38, 150.94,115.61, 68.61, 62.66, 62.53, 61.92,49.77, 31.57, 29.26, 25.68, 22.57, 20.53, 20.29, 14.01.

**1-[2-4-(heptyloxy)phenoxy)ethyl]-1-methylpiperidin-1-ium (12b)**

Brown liquid; ^1^H NMR (400 MHz, CD_3_OD) δ 6.99 (d, *J* = 9.1 Hz, 2H), 6.91 (d, *J* = 9.1 Hz, 2H), 3.96 (t, *J* = 6.4 Hz, 3H), 3.92 (m, 2H), 3.59 (tdd, *J* = 18.4, 12.5, 5.6 Hz, 4H), 3.29 (s, 3H), 2.02 (s, 4H), 1.77 (dd, *J* = 12.1, 5.8 Hz, 4H), 1.44 (m, 10H), 0.96 (t, *J* = 6.5 Hz, 3H). ^13^C NMR (101 MHz, CD_3_OD) δ 155.64, 152.93, 116.82, 116.60, 69.58, 63.78, 63.41, 54.82, 32.96, 30.46, 30.19, 27.12, 24.02, 23.65, 22.53, 21.94, 21.09, 14.40.

**1-methyl-1-(2-(4-(octyloxy)phenoxy)ethyl)piperidin-1-ium (12c)**

Brown liquid; ^1^H NMR (400 MHz, CD_3_OD) δ 6.85 (d, *J* = 9.0 Hz, 2H), 6.76 (d, *J* = 8.9 Hz, 2H), 3.81 (t, *J* = 6.4 Hz, 2H), 3.78 (m, 2H), 3.44 (m, 4H), 3.14 (s, 3H), 1.84 (m, 4H), 1.62 (m, 4H), 1.28 (m, 12H), 0.80 (t, *J* = 6.2 Hz, 3H). ^13^C NMR (101 MHz, CD_3_OD) δ 155.62, 152.13, 116.83, 116.59, 69.57, 63.80, 63.41, 63.36, 54.82, 32.96, 30.47, 30.45, 30.37, 27.15, 24.02, 23.69, 22.52, 21.93, 21.09, 14.43.

**Spectral Characterization of Products**

**Supplementary Figure 1.** NMR ^1^H spectrum of compound (**11a**) (CDCl_3_, 400 MHz)

**Supplementary Figure 2.** NMR ^13^C spectrum of compound (**11a**) (CDCl_3_, 101 MHz)

**Supplementary Figure 3.** NMR ^1^H spectrum of compound (**11b**) (CDCl_3_, 400 MHz)

**Supplementary Figure 4.** NMR ^13^C spectrum of compound (**11b**) (CDCl_3_, 101 MHz)

**Supplementary Figure 5.** NMR ^1^H spectrum of compound (**11c**) (CDCl_3_, 400 MHz)

**Supplementary Figure 6.** NMR ^13^C spectrum of compound (**11c**) (CDCl_3_, 101 MHz)

**Supplementary Figure 7.** NMR ^1^H spectrum of compound (**12a**) (CDCl_3_, 400 MHz)

**Supplementary Figure 8.** NMR ^13^C spectrum of compound (**12a**) (CDCl_3_, 101 MHz)

**Supplementary Figure 9.** NMR ^1^H spectrum of compound (**12b**) (CD_3_OD, 400 MHz)

**Supplementary Figure 10.** NMR ^13^C spectrum of compound (**12b**) (CD_3_OD, 101 MHz)

**Supplementary Figure 11.** NMR ^1^H spectrum of compound (**12c**) (CD_3_OD, 400 MHz)

**Supplementary Figure 12.** NMR ^13^C spectrum of compound (**12c**) (CDCl_3_, 101 MHz)

**Supplementary Figure 13.** **RMSD** of Cα atoms fluctuated over 4.0 Å during the simulation processes to **11a**/α7 complex.

**Supplementary Figure 14. RMSD** of Cα atoms fluctuated over 3.0 Å during the simulation processes to **12a**/α7 complex.


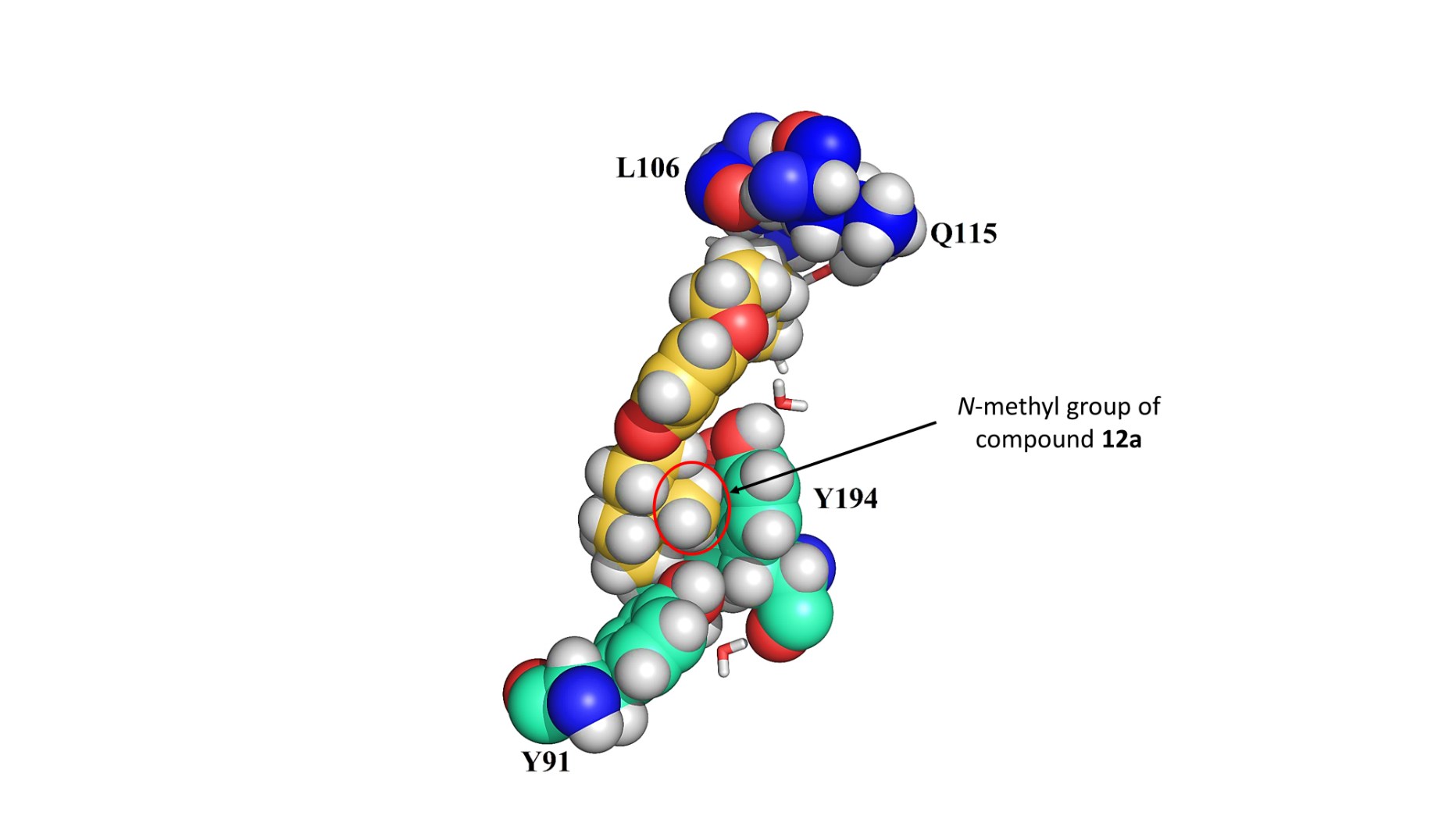


**Supplementary Figure 15***. N*-methyl group of **12a** interacts with Y194.

**
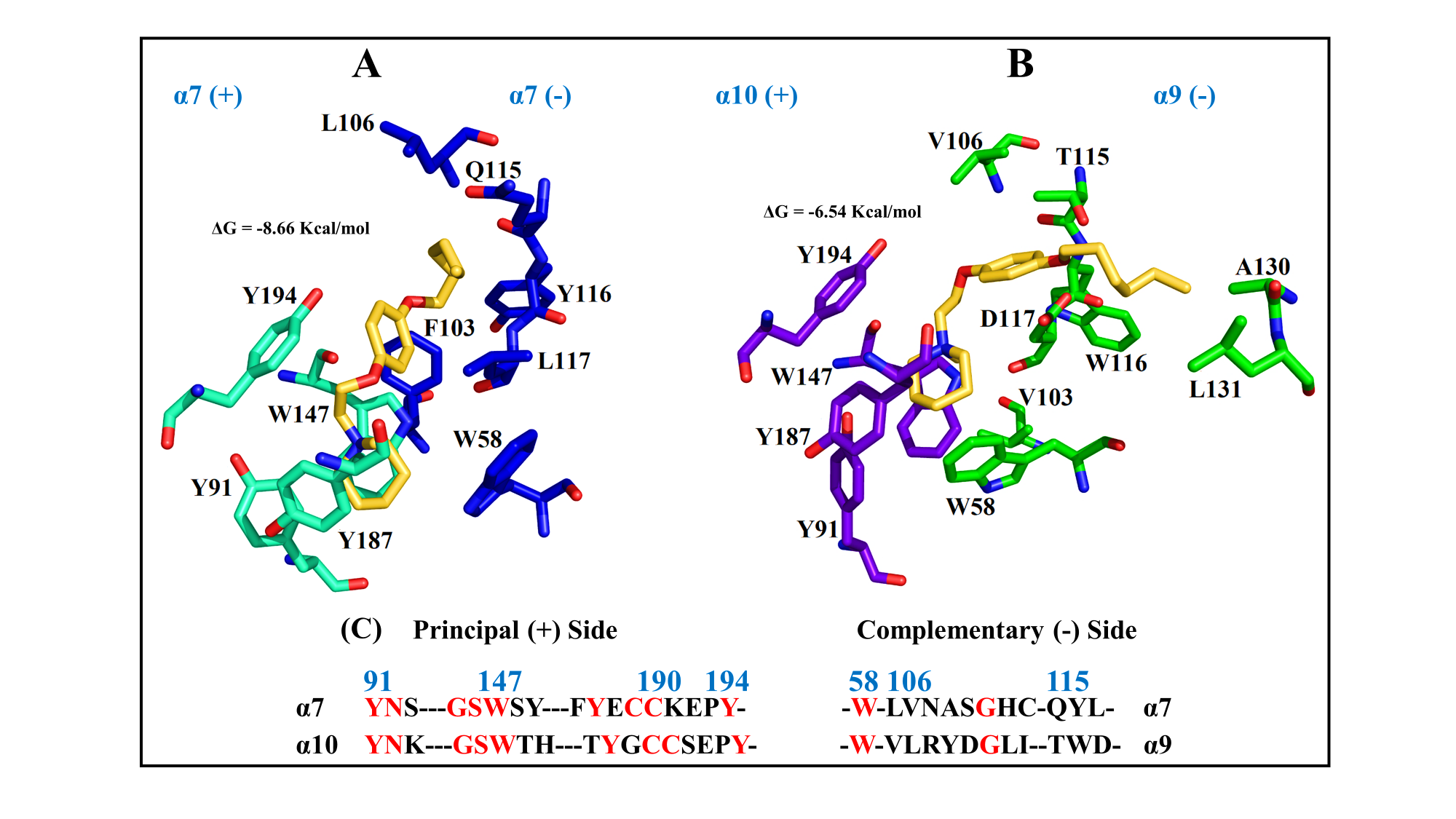
**

**Supplementary Figure 16**. **(A)** Molecular docking at the rat α7 nAChR active site for 12a (in yellow). Amino acid residues forming the principal (+)-side are colored in cyan, and those forming the complementary (-)-side are in blue according to the convention used for the X-ray crystal structure of *Lymnaea stagnalis* AChBP (Celie et al., 2004). **(B)** Molecular docking at the rat α9α10 nAChR for 12a. Amino acid residues forming the principal (+)-side are colored in purple, and those forming the complementary (-)-side are in green. **(C)** Alignments of the amino acid sequences of the α7, α9 and α10 subunits.
